# A novelty-retrieval-extinction paradigm leads to persistent attenuation of remote fear memories

**DOI:** 10.1038/s41598-020-60176-2

**Published:** 2020-02-24

**Authors:** Fulian Huang, Guangjing Zou, Can Li, Hui Meng, Xiaoyan Liu, Zehua Yang

**Affiliations:** 1 Department of Physiology, Yiyang Medical College, Yiyang, Hunan 413000 China; 2 Department of Anatomy and Neurobiology, School of Basic Medical Science, Central South University, Changsha, Hunan 410013 China

**Keywords:** Epigenetics and behaviour, Extinction

## Abstract

Exposure to a novel environment can enhance the extinction of recent contextual fear in mice. This has been explained by a tagging and capture hypothesis. Consistently, we show in mice that exposure to a novel environment before extinction training promoted the extinction of recent auditory fear. However, such a promoting effect of novelty was absent for remote memories. In the present study, we replaced the regular extinction training with a retrieval-extinction session which capitalized on a reconsolidation window. When novelty exposure was followed by a retrieval-extinction session, remote fear was distinguished more easily and permanently. We have termed it as a “novelty-retrieval-extinction” paradigm. This paradigm played a greater role in the extinction of remote fear when fear conditioning and retrieval-extinction occurred in two different contexts other than in one identical context. The mechanism underlying the facilitating effect of this paradigm might involve up-regulation of histone acetylation in the hippocampus, which has been reported to increase functional and structural neuroplasticity. The present work proposes an effective, drug-free paradigm for the extinction of remote fear, which could be easily adapted in humans with least side effects.

## Introduction

Post-traumatic stress disorder (PTSD) is generally characterized by an intense and enduring memory for the traumatic events^1,2^. Presently, there is considerable agreement that exposure-based therapy for PTSD is highly analogous to fear extinction^3,4^, during which fear inhibition is achieved by repeated exposure to the fear-inducing stimulus in the absence of any aversive event^4^.

Studies in both animals and humans have proved that a reconsolidation-updating paradigm can permanently attenuate recent fear memories by presenting extinction training within a reconsolidation window opened by an isolated conditioned stimulus (CS)^5,6^. Reconsolidation occurs when a consolidated memory is retrieved and returns to a labile and protein synthesis-dependent state. According to this paradigm, extinction training applied within the reconsolidation window incorporates the information that the once fear-inducing CS is safe into an updated memory. However, this paradigm failed to attenuate remote fear memories efficiently^7,8^. The reason may be that although recent memory recall led to increased S-nitrosylation of histone deacetylase 2 (HDAC2) and acetylation of histone 3 on lysine residues 9 and 14 (H3K9/14), remote memory recall did not induce histone acetylation-mediated neuroplasticity^8^. Inspiringly, by administrating of HDAC2 inhibitor (HDACi), the reconsolidation-updating paradigm reinstates hippocampal neuroplasticity and permanently extinguishes remote fear memories^8^. However, HDACis are not suitable to apply to humans.

In this study, we modified the reconsolidation-updating paradigm, with a process known as behavioural tagging, to permanently attenuate remote fear using behavioural interventions alone.

Behavioural tagging, which stems from the synaptic tagging and capture theory^9–12^, suggests that memory persistence can be facilitated by a second behavioural event which subsequently induces the synthesis of required proteins^10–12^. For example, exposure to a novel environment facilitated contextual fear conditioning^13^, fear extinction^14,15^, conditioned taste memory^13^, object recognition^13^, and inhibitory avoidance^16^. Hypothetically, exploration of a novel open field (OF) triggers the synthesis of plasticity-related proteins (PRPS) that can be captured by a learning tag set by a weak training and leads to the establishment of a persistent mnemonic trace^11,16^. Considering the potential synergy of behavioral tagging and updating of reconsolidation, in the present work, we studied whether the extinction of remote fear memory can be facilitated by novelty and whether the extinction memory persists, presumably through an up-regulation of H3K9/14 acetylation.

## Results

### Effects of novelty on extinction of recent and remote auditory fear

First, mice were trained in an auditory fear conditioning task consisting of five CS-US pairs. Twenty-four hours or 30 days later, mice underwent an extinction training session to investigate if recent and remote fear extinction can be promoted by a novelty exposure to an OF. We employed a weak extinction training session that included 12 CSs, in contrast to 20 CSs used in other studies^5,17^. Twenty-four hours after extinction training, extinction test was performed to evaluate long-term memory (LTM) of extinction.

**Figure 1:**
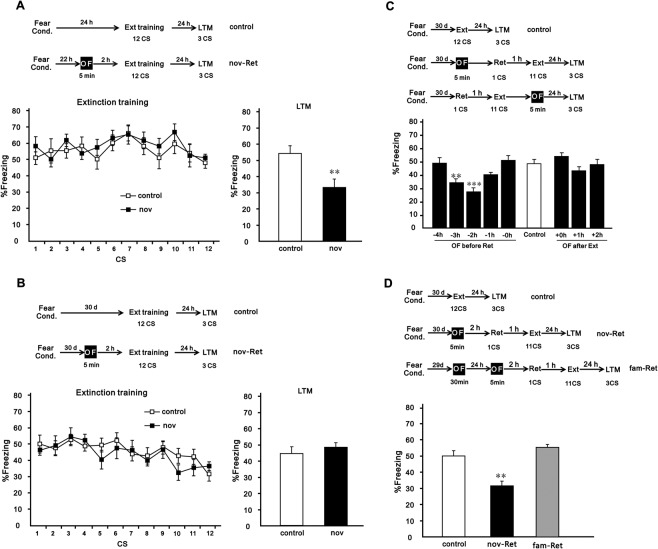
Effects of novelty on extinction of recent and remote auditory fear. (**A**,**B**) Mice were fear conditioned with five tone-shock pairings. After 24 hours (**A**) or 30 days (**B**), they were trained in a weak extinction training task with 12 CSs. Mice were exposed to a novel OF for 5 min 2 h before extinction training (nov), or left unexposed (control). In all cases mice were submitted to an LTM test session with 3 CSs, 24 h after the extinction training session. For recent memory: nov, n = 7, control, n = 7; for remote memory: nov, n = 9, control, n = 10. (**C**) Effects of novelty at various intervals either before or after the retrieval-extinction training session. The LTM test performance of mice is shown. Thirty days after fear conditioning, mice received an extinction training session as mentioned before. During the extinction session, control mice received only a regular extinction procedure and were not exposed to the OF or retrieval session. Other mice received a retrieval-extinction session and were exposed to the OF for 5 min 4 h, 3 h, 2 h, 1 h or 0 h before the retrieval session or 2 h, 1 h, or 0 h after extinction training session. n = 10 for -4 h group and n = 9 other groups. (**D**) Familiar OF had no effects on the extinction of remote fear memory. fam-Ret, n = 8; nov-Ret, n = 8. Data are presented as mean ± SEM of the percentage of time spent freezing. *P < 0.05, **P < 0.01, ***P < 0.01 *vs*. control group. OF, open field; LTM, long-term memory; CS, conditioned stimuli; SEM, standard error of mean.

All mice acquired equivalent conditioned fear to the auditory CSs (see Supplementary Fig. S1). Mice were either exposed to a 5-min OF session 2 h before the extinction training session or left unexposed (Fig. 1). The mice were allowed to explore the OF freely, then returned to their home cages^13–16^. Figure 1A,B show no significant effect of group during both recent and remote extinction training sessions (*F* (1, 12) = 0.941, *p* = 0.351 and *F* (1, 17) = 0.334, *p* = 0.571, respectively). The test for LTM of extinction showed that exposure to the OF 2 h before the extinction session significantly enhanced the extinction of recent fear memory (*t* (12) = 3.116, *p* = 0.009) (Fig. 1A). This result is consistent with a previous study on the effect of novelty on recent contextual fear extinction^14^. However, the enhancing effect of exposure to the OF was not seen in the extinction of remote fear (*t* (17) = 0.806, *p* = 0.431) (Fig. 1B). Intriguingly, when regular extinction training was replaced with a reconsolidation-updating paradigm, exposure to the OF 2 h before the retrieval session significantly enhanced the extinction of remote fear memory (Fig. 1C).

It was reported that novelty exposure 1 h or 2 h before the extinction training session, or 1 h after extinction significantly enhanced contextual fear extinction^14^. In this study, mice were exposed to an OF for 5 min at various intervals either before or after the retrieval-extinction training session, or were unexposed before the extinction training (control group) (Fig. 1C). The control mice were exposed to regular extinction training because mice froze equally during both extinction training (*F* (1, 14) = 2.147, *p* = 0.165) and LTM test (*t* (14) = 1.059, *p* = 0.308) even if they were exposed to a conditioning context (no-Ret) or simply left in the home cage (control) before extinction training (see Supplementary Fig. S2). All groups showed similar freezing during extinction training (*F* (8, 73) = 0.464, *p* = 0.878) (see Supplementary Fig. S3). LTM test indicated that exposure to the OF 2 h or 3 h before the retrieval session significantly enhanced extinction compared with the control group (main group effects: *F* (8, 73) = 5.87, *p* < 0.001; *post hoc* comparisons: −2 h, *p* < 0.001 and −3 h, *p* = 0.039, *vs*. control)(Fig. 1C). Exposure to the OF at other time points before the retrieval session or at all time points after extinction had no effects (−4 h, *p* = 0.999; −1 h, *p* = 0.428; −0 h, *p* = 0.998; +0 h, *p* = 0.797; +1 h, *p* = 0.82; +2 h, *p* = 0.999, *vs*. control) (Fig. 1C).

However, the enhancing effect of exposure to the OF before the retrieval-extinction training session was not seen when the OF was familiar. As shown in Fig. 1D, mice were exposed to the OF for 30 min one day before extinction (fam-Ret group). Subsequent exposure of the fam-Ret group to the same OF before retrieval-extinction, indicated familiarity rather than novelty. During extinction training, the fam-Ret, nov-Ret, and control groups froze equally (*F* (2, 21) = 0.398, *p* = 0.677) (see Supplementary Fig. S4). LTM test indicated that exposure to a novel OF 2 h before retrieval-extinction significantly enhanced extinction (main group effects: *F* (2, 21) = 19.44; *post hoc* comparisons: nov-Ret, *p* < 0.001 *vs*. control), but exposure to a familiar OF had no effects (fam-Ret, *p* = 0.336 *vs*. control) (Fig. 1D). These results were consistent with that reported by a previous study^14^.

### Exposure to an OF before retrieval-extinction session prevents spontaneous recovery, renewal and reinstatement of remote auditory fear

**Figure 2:**
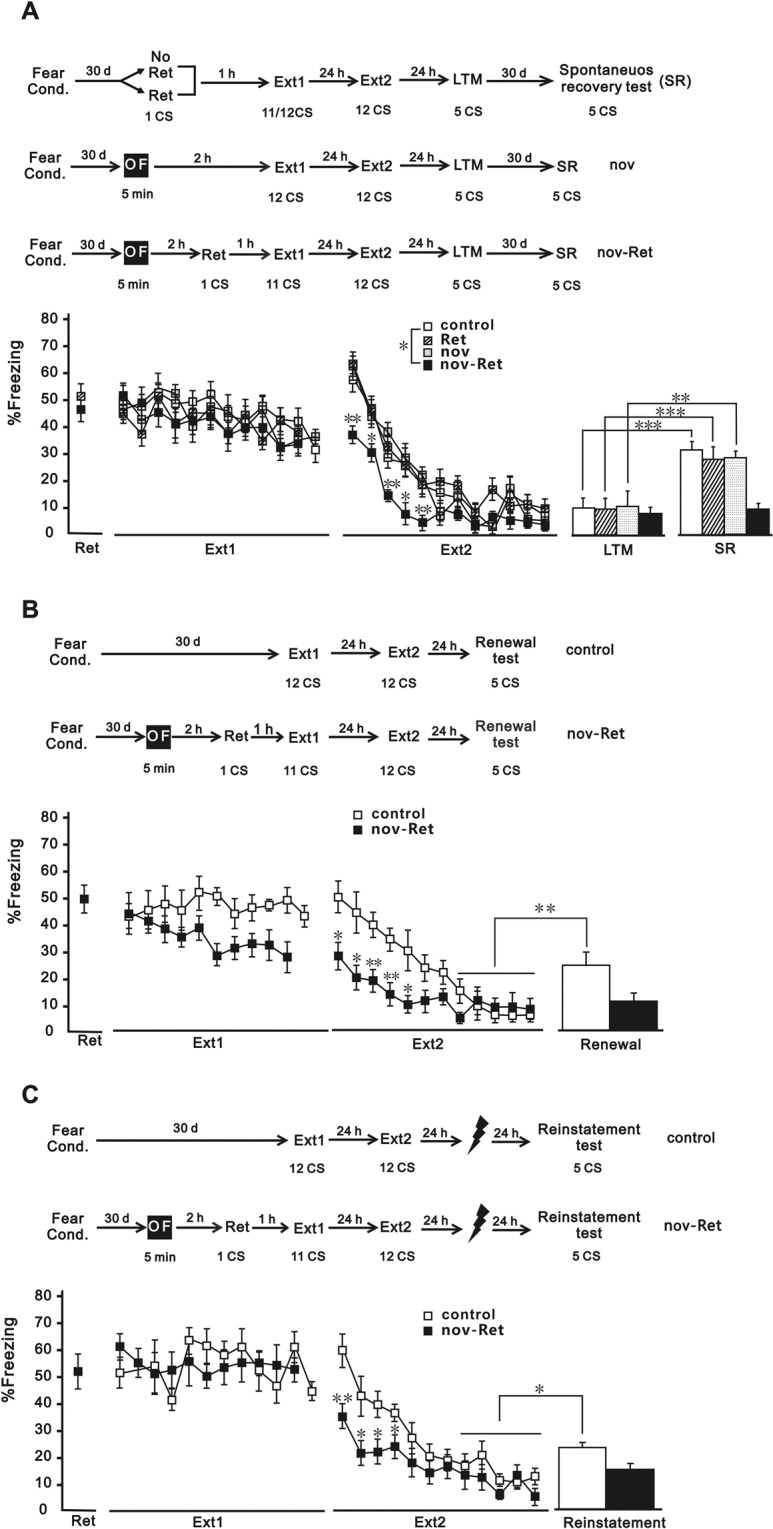
Exposure to an OF before retrieval-extinction session prevents the return of remote auditory fear. (**A**) Mice were fear conditioned. Thirty days after fear conditioning, they were exposed either to a regular extinction training session (control/no Ret, n = 9), or a retrieval-extinction session (Ret, n = 9), or an OF session followed by a regular extinction training session (nov, n = 10) or by a retrieval-extinction session (nov-Ret, n = 9). Twenty-four hours later, all groups received a second extinction training with 12 CSs to ensure that all groups froze equivalently at the end of extinction. Twenty-four hours after extinction, all groups were tested for LTM with 5 CSs, and 1 month later for spontaneous recovery with 5 CSs. (**B**) Mice were fear conditioned in context A. Thirty days after fear conditioning, they were exposed either to a regular extinction training session (control, n = 11) in context B, or an OF session followed by a retrieval-extinction session (nov-Ret, n = 12) in context B. Twenty-four hours later, all groups received a second extinction training in context B. Twenty-four hours after extinction, all groups were tested for renewal with 5 CSs in context A. (**C**) Twenty-four hours after extinction, both the control group (n = 8) and nov-Ret group (n = 11) received five un-signalled foot-shocks, and the next day they were tested for reinstatement with 5 CSs. Data are presented as mean ± SEM of the percentage of time spent freezing. *P < 0.05, **P < 0.01, ***P < 0.01 *vs*. control group. OF, open field; LTM, long-term memory; CS, conditioned stimuli; SEM, standard error of mean.

To investigate whether our procedure could prevent the return of fear, we examined its effect on three assays: spontaneous recovery, renewal, and reinstatement of fear; the presence was indicative of incomplete fear attenuation^18–22^. The mice exposed for 5 min to an OF 2 h before the retrieval-extinction training session showed no signs of spontaneous recovery, renewal or reinstatement of the fear (Fig. 2), indicating persistence of the extinction memory.

For the spontaneous recovery experiment, all procedures were conducted in context A. As shown in Fig. 2A, during the first extinction training phase, all groups exhibited a similar fear response (*F* (3, 33) = 0.556, *p* = 0.648). During the second extinction training phase, there was a significant effect of group (*F* (3, 33) = 6.093, *p* = 0.002). Post hoc comparisons showed both the nov and Ret groups presented equal freezing to CS, indicating that neither an OF session nor a retrieval session facilitated the extinction of remote memory (nov, *p* = 0.999, ret, *p* = 0.81, *vs*. control). However, the nov-Ret group showed lower freezing than the control group (*p* = 0.009, *vs*. control), indicating the facilitating effects of the procedure. All groups performed equivalently during the LTM test (*F* (3, 33) = 0.79, *p* = 0.51). One month later, the control, nov, and Ret groups showed increased freezing (spontaneous recovery) relative to their respective freezing in the LTM test [control, *t* (16) = 6.195, *P* < 0.001; nov, *t* (16) = 3.903, *P* = 0.001; Ret, *t* (18) = 5.955, *P* < 0.001]; however, the nov-Ret group did not show this pattern (*t* (16) = 0.565, *P* = 0.58).

Surprisingly, when the extinction context was different from the context for fear conditioning as in the experiment to assess fear renewal (Fig. 2B), the nov-Ret group presented significantly lower level of freezing response than the control group during first extinction training sessions (*F* (1, 21) = 4.364, *p* = 0.049). Moreover, during the second extinction training session, the nov-Ret group showed a lower level of freezing (*F* (1, 21) = 5.559, *p* = 0.28). Both groups froze equivalently to the last 5 CSs of extinction. When placed back in the fear acquisition context, increased freezing was observed in the control group (fear renewal; *t* (20) = 2.955, *P* = 0.008), but not in the nov-Ret group (*t* (22) = 0.647, *P* = 0.525), relative to their respective last 5 CSs of extinction.

For the fear reinstatement experiment, all procedures were conducted in context A (Fig. 2C). The control and nov-Ret groups froze equivalently during the first extinction session (*F* (1, 17) = 0.001, *p* = 0.981). During the second extinction session, the nov-Ret group showed reduced freezing compared with the control group (*F* (1, 17) = 4.74, *p* = 0.44). Fear reinstatement was tested 24 hours after 5 un-signalled foot-shocks. The control group showed increased freezing (fear reinstatement; *t* (14) = 2.452, *P* = 0.028), but not the nov-Ret group (*t* (20) = 1.161, *P* = 0.259), relative to their respective last 5 CSs of extinction.

### Exposure to an oF before retrieval-extinction session increased hippocampal H3K9/14 acetylation

**Figure 3:**
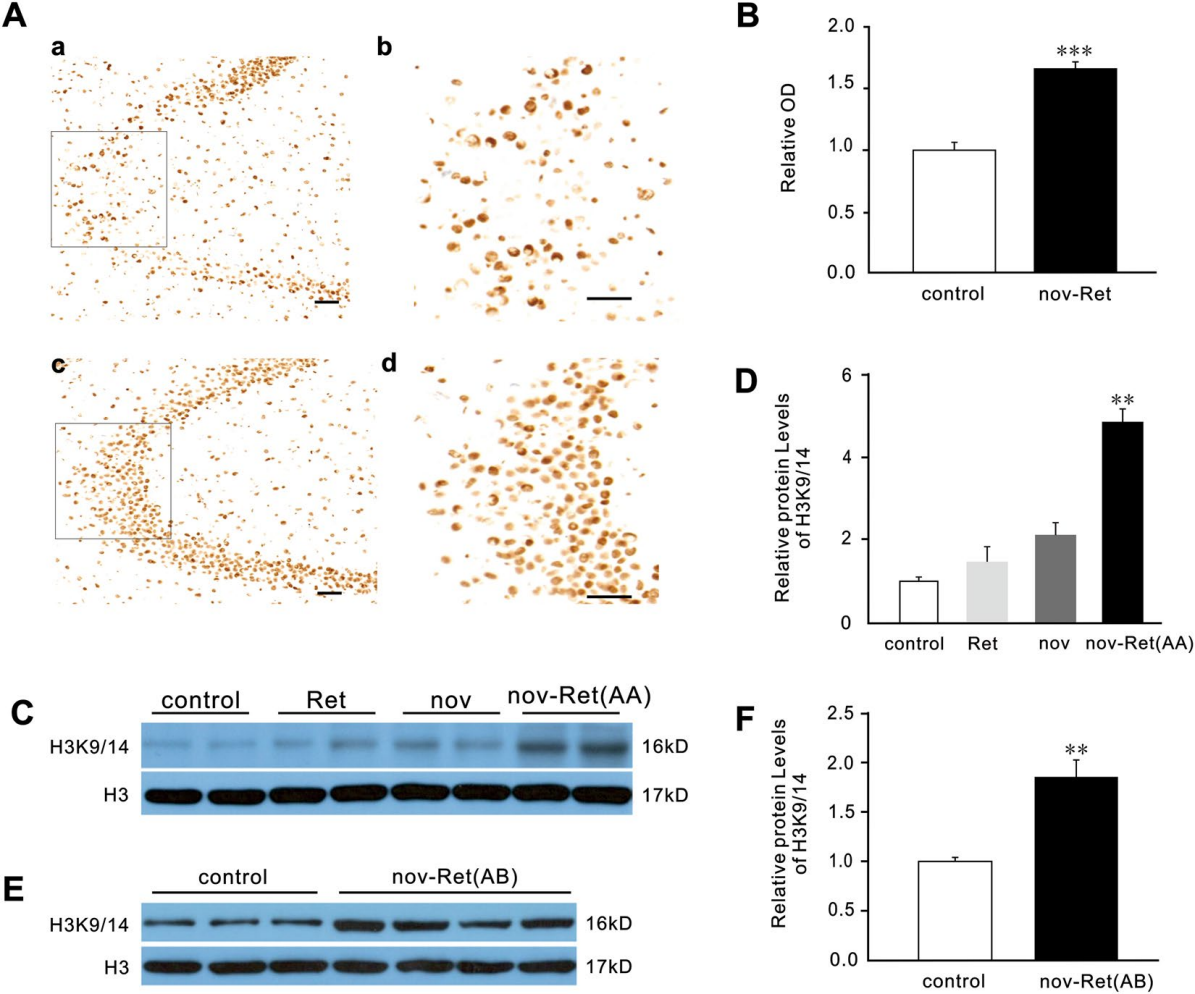
Exposure to an OF before the retrieval-extinction session increased hippocampal H3K9/14 acetylation. (**A**) Representative images showing immunostaining for H3K9/14ac in the hippocampal CA3 region 1 h after completion of extinction in nov-Ret and control mice. Higher magnification images of H3K9/14ac staining in the CA3 are shown for the control (b) and nov-Ret groups (d) and correspond to the labelled regions in a and c, respectively. Scale bar: 100 μm. (**B**) Immunohistochemical analysis of OD for H3K9/14ac in the hippocampus for each group. n = 4 mice in each group. (**C**,**E**) Pictures of western blotting analysis of H3K9/14ac in the hippocampus 1 h after completion of extinction in control and nov-Ret mice. control, n = 5; Ret, n = 2; nov, n = 2; nov-Ret, n = 6. Two mice in the nov-Ret group received fear conditioning and retrieval-extinction in the same context (AA), and another four in different contexts (AB). Original western blotting images are shown in Supplementary Fig. S6. (**D**,**F**) Densitometric analysis for H3K9/14ac in (**C**,**E**), respectively. Data are presented as mean ± SEM. **P < 0.01, ***P < 0.001 *vs*. control. OF, open field; SEM, standard error of mean.

Increments in H3K9/14 acetylation played a role in inducing neuronal plasticity in the hippocampus and in facilitating the update of remote memories^8^. To test whether exposure to an OF before retrieval-extinction session increases hippocampal H3K9/14 acetylation, we assessed the acetylation of H3K9/14. Immunohistochemical analyses showed that 1 h after extinction training, hippocampal H3K9/14 acetylation of the nov-Ret group was significantly higher than that of control animals (*t* (6) = 7.108, *P* < 0.001) (Fig. 3A,B), but was indistinguishable in the prefrontal cortex and amygdala (*t* (6) = 0.677, *P* = 0.524; *t* (6) = 0.291, *P* = 0.781, respectively) (see Supplementary Fig. S5). Western blotting analyses confirmed these results, and showed that 1 h after extinction training, H3K9/14 acetylation in the hippocampus of the nov-Ret group was significantly higher than that of control animals (main group effects: *F* (3, 4) = 36.25, *p* = 0.002; *post hoc* comparisons: nov-Ret, *p* = 0.002 *vs*. control) (Fig. 3C,D), but it was comparable between the control group and the Ret group or nov group (Ret, *p* = 0.594 and nov, *p* = 0.115, *vs*. control). Furthermore, the results showed that our procedure increase hippocampal H3K9/14 acetylation irrespective of whether fear conditioning and retrieval-extinction occured in the same context (nov-Ret (AA), *p* = 0.002, *vs*. control) (Fig. 3C,D) or in different contexts (nov-Ret (AB), *t* (5) = 4.04, *P* = 0.0099, *vs*. control)(Fig. 3E,F).

## Discussion

Behavioural tagging proposes that a weak learning event can set a tag that could capture the PRPs produced by a strong learning event. For example, exposure to a novel environment facilitated recent contextual fear extinction^14^. Our present results show that novelty exploration promoted recent auditory fear extinction further supporting this hypothesis (Fig. 1A). However, the promoting effect of novelty was not found in the extinction of remote auditory fear (Fig. 1B). This result is easy to interpret because remote memories are more stable than recent ones^23–26^ and refractory to behavioural attenuation^27^. Interestingly, novelty facilitated the extinction of remote fear when extinction training was performed during the reconsolidation window (Fig. 1C), in which reactivated memories are amenable to enhancement or disruption. Furthermore, novelty exploration in combination with the reconsolidation-updating paradigm attenuate remote fear permanently because there were no signs of spontaneous recovery, renewal, or reinstatement of fear (Fig. 2), indicators of incomplete fear extinction^18–22^. These data suggest that the original fear memory is no longer intact, which is consistent with an update during reconsolidation. Instead of novelty exploration, these facilitatory effects on remote extinction can be achieved by using HDACi, CI-994, in combination with extinction training^8^.

Although the reconsolidation-updating paradigm can lead to persistent attenuation of recent fear, previous studies^7,8^ and the results of our present study (Fig. 2A) showed that it failed to permanently diminish remote fear. Graff *et al*.^8^ identified that the lack of histone acetylation-mediated neuroplasticity in the hippocampus was one potential mechanism to prevent the attenuation of remote fear, which can be enhanced by using CI-994 in combination with extinction training. In our study, we replaced the pharmacological intervention in the previous study with a pure behavioural alternative, which we refer to as the novelty-retrieval-extinction paradigm. Intriguingly, we observed up-regulation of acetylated H3K9/14 (H3K9/14ac) in the hippocampus (Fig. 3), an effect similar to CI-994 treatment, which resulted in increased functional and structural neuroplasticity^8^. Thus, though presently undetermined, the use of our paradigm may enhance hippocampal neuroplasticity necessary for remote extinction. Nevertheless, the mechanisms of novelty exploration leading to H3K9/14acetylation in the context of memory recall merits further investigation.

Our results further suggest that our novelty-retrieval-extinction paradigm played a greater role in the extinction of remote fear when fear conditioning and extinction occurred in two different contexts than in one identical context (Fig. 2B). As shown in the results, despite facilitated between-session extinction, facilitated within-session extinction was observed only when fear conditioning and extinction occurred in different contexts (Fig. 2B), and not when these procedures occurred in the same context (Fig. 2A,C).However, the expression of H3K9/14ac increased in both paradigms (Fig. 3), suggesting that H3K9/14ac does not contribute to the regulation of within-session extinction. Although within- and between-session extinctions differ at the molecular and anatomical levels^28^, it is preferable to clarify the mechanism underlying the discrepant effects of our paradigm on within- and between-session extinctions.

Behavioural tagging studies have shown that brief exploration in a novel OF can facilitate weak learning in various types of memory such as spatial object recognition, conditioned taste aversion learning tasks^13^ and fear extinction^14^, regardless of whether the novel exploration is employed before or after the weak event, as described previously. For example, exposure to novelty can facilitate the extinction of recent contextual fear whether it is introduced before or after the extinction training^14^. However, this symmetric feature in timing was not seen in the extinction of remote auditory fear in the present study (Fig. 1C). One cause may be that the reconsolidation-extinction session in our present research lasts about one hour and twenty minutes while the tag had a limited duration^10^. Moreover, the time windows of efficacy for tagging and capture processes to occur are different. This process of environmental exploration is effective within a restricted time window of approximately 1–2 h before or after the weak training^13–15^. However, a novelty exposure session too close to the weak event might have a negative effect^29^. The difference in temporal symmetry and time window may suggest multiple routes to modulating memory^30^.

As traumatic memories are persistent and often not readily available for early interventions^31^, there is a clear need to find effective treatment options for remote trauma. Through the interpolation of novelty, the present work devised an effective, drug-free paradigm for the facilitated and persistent reduction of remote fear, which should be easy to apply in humans with minimal side effects.

## Material and Methods

### Animals

Adult male C57BL∕6 mice (9–17 weeks), purchased from the Laboratory Animal Center of Central South University, Changsha, China, were used. They were housed in groups of 3–4 per cage, with *ad libitum* access to food and water, under a 12 h light/dark cycle (lights on at 7 AM) at a room temperature of 20–25 °C. Before all behavioural procedures, the mice were handled 3 min daily for 3 days to eliminate handling stress as a confounding variable. Experiments were conducted according to the *National Institutes of Health Guide for the Care and Use of Laboratory Animals*, and experimental protocols were approved by the animal care and use committee of Central South University.

### Behavioural apparatus

Conditioning chambers. Mice were trained and tested in fear conditioning chambers (Huaibei Zhenghua Biological Equipment Co. Ltd., Anhui, China), as described previously (Fulian Huang, BMC Neuroscience 2014, 15:86). The chambers (23 cm × 23 cm × 32 cm, without ceiling) were situated within a sound-attenuating cabinet individually. For context A, the walls of the chamber were made of white opaque acrylic boards, and the floor consisted of 23 stainless steel bars spaced 10 mm apart that were connected to a shock generator and scrambler for the delivery of foot-shock USs. The presentation and sequencing of all stimuli was controlled by a custom written computer program. The chamber was thoroughly cleaned with water and dried between sessions. Context B consisted of black opaque acrylic walls and a white opaque acrylic floor. The chamber was thoroughly cleaned with 75% ethanol and dried between sessions.

OF box. The OF was an open-top square box (46 × 46 × 46 cm) made of opaque Plexiglas walls and wooden floor with waterproof coating. It served as a novel environment when mice were exposed to the OF for 5 min, while it served as a familiar environment when mice were exposed to the OF for 30 min 24 h before they were exposed to the OF again^14^.

### Cued fear conditioning and extinction

The behavioral training was performed as described previously (Fulian Huang, BMC Neuroscience 2014, 15:86). Mice were habituated to the conditioning chamber (context A) for 10 min with no stimuli presented one day before fear conditioning. During fear conditioning phase, the mice were allowed to explore the chamber for 3 min. After 3 min, they were subjected to five trials of audio tone (CS) and foot-shock (US) with an inter-trial interval (ITI) of 90 s. Audio tone (4 kHz, 80 dB, 20 s duration) was followed immediately by a foot-shock (0.5 mA, 1 s duration) from the metal grid floor. The mice remained in the training box for 60 s following the last CS-US pairing, after which they were returned to the home cages.

In experiments shown in Fig. 1A,B, 1 day or 30 days after fear conditioning, mice received an extinction training session consisting of 12 CSs with an ITI between CSs of 60 s. Twenty-four hours later, they underwent an LTM test (3 CSs) to assess the LTM of extinction.

In other experiments, 30 days after fear conditioning, mice received either a retrieval trial (Ret and nov-Ret), or were left unexposed (control and nov), or exposed to the conditioning context (no-Ret). One hour later—during which time the mice were returned to their home cage—they received an extinction training session consisting of 11 or 12 CSs with an ITI between CSs of 60 seconds. Mice in the retrieval group (Ret and nov-Ret) received 11 CSs, and those in unexposed group (control and nov) received 12, ensuring that each group was presented with the same number of CSs. Mice received one or two extinction training sessions, followed by an LTM test (3 or 5 CSs), depending on the experiment; 3 CSs were used when they received one extinction training session to better manifest the group effect; 5 CSs were used when they received two extinction training sessions, for the definite effect of fear extinction.

Spontaneous recovery: Thirty days after extinction, mice were tested for spontaneous recovery of fear by exposure to 5 CSs.

Renewal: For the renewal experiment, mice were fear conditioned in context A, then retrieved and extinguished in context B. Twenty-four hours after extinction, the mice were tested for renewal of fear in context A by exposure to 5 CSs. For other experiments, all procedures took place in context A.

Reinstatement: Twenty-four hours after extinction, mice received five 1-s foot-shocks (0.5 mA) with an ITI of 30 s, after a 20-s habituation period (without tone) to the chamber. Twenty-four hours later, they were tested for reinstatement of fear by exposure to five tones.

Freezing was used as the measure of the conditional fear response and expressed as a % value from total CS. Freezing is characterized by cessation of movement except that required for respiration. The total time spent freezing during every 20-s tone CS was scored offline using a digital stopwatch based on digital videos. Observers scoring the freezing were blinded to the treatments.

### Immunohistochemistry

Immunohistochemistry was performed as described previously (Hong-Tao Wang, Neurotox Res 2017, 31:505–520). Coronal brain slices (25 µm thickness) were incubated at room temperature in a blocking buffer solution containing 5% bovine serum and 0.1% Triton X-100 in 0.01 M PBS. Sections were then incubated overnight at 4 °C with rabbit anti-H3K9/14ac (1:1000, Origene, TA347180). The sections were washed with a 0.1% Triton X-100 solution in 0.1 M PBS and further incubated with the VECTASTAIN ABC Kit (Vector Laboratories, USA, PK-4001). Diaminobenzidine tetrahydrochloride (1:2000, Sigma-Aldrich, St. Louis, MO, USA, D5905) was used as a peroxidase substrate. Images were quantified using HPIAS-1000 image analysis by an experimenter blinded to the treatment groups.

### Western blotting analysis

Western blotting analysis was performed as reported previously (Hong-Tao Wang, Neurotox Res 2017, 31:505–520). The hippocampi were removed and snap-frozen in liquid nitrogen. Nuclear extracts were collected using Nuclear and Cytoplasmic Extraction Reagent Kit (Beyotime Biotechnology, China) according to the manufacturer’s protocol. Thirty micrograms of protein from each sample was loaded onto and separated by a 15% Bis-Tris Sodium Dodecyl Sulfate PolyAcrylamide Gel Electrophoresis gel. The blotted proteins were transferred onto nitrocellulose membranes. The membranes were blocked with 5% non-fat milk for 2 h at room temperature and incubated overnight with the following antibodies: rabbit polyclonal anti-histone H3 antibody (Servicebio; dilution, 1:30000) and rabbit polyclonal antibody H3K9/14ac (Origene; dilution, 1:1000). Then, the membranes were washed and incubated with horse-raddish peroxidase conjugated secondary antibodies (CWBIO; dilution, 1:1000) for 2 h at room temperature. After three rinses (10 min in each) in tris-buffered saline with 0.1% Tween-20, immunoreactivity was detected with an ECL Western Blotting Detection Kit (CWBIO, China). Results were standardized to histone H3 control protein.

### Statistical analyses

Statistical analyses were performed using software SPSS (Version 13; SPSS, Chicago, IL). Significant differences between the two groups were determined using Student’s t tests. One-way or two-way analysis of variance with the Tukey honest significant difference method was used for multiple comparisons. All data were represented as mean ± SEM. Significant level was set at *P* < 0.05.

## Supplementary information

Supplementary Figures.

## Data Availability

The data that support the findings of this study are available from the corresponding author upon request.
