# Supplementary material for: A novelty-retrieval-extinction paradigm leads to persistent attenuation of remote fear memories
Source: Sci Rep. 2020 Feb 24;10:3319. doi: 10.1038/s41598-020-60176-2 (PMC7039928; doi:10.1038/s41598-020-60176-2)
Supplement: Supplementary file 1 — Supplementary Figures. [file 41598_2020_60176_MOESM1_ESM.docx]

Supplementary Information

A novelty-retrieval-extinction paradigm leads to persistent attenuation of remote fear memories

Fulian Huang^1*^, Guangjing Zou^2^, Can Li^1^, Hui Meng^1^, Xiaoyan Liu^1^, Zehua Yang ^1*^

^1^ Department of Physiology, Yiyang Medical College, Yiyang, Hunan 413000, China; ^2^ Department of Anatomy and Neurobiology, School of Basic Medical Science, Central South University, Changsha, Hunan 410013, China.

^*^ To whom correspondence may be addressed. E-mail: hflscience@126.com or ouyang7575737@126.com


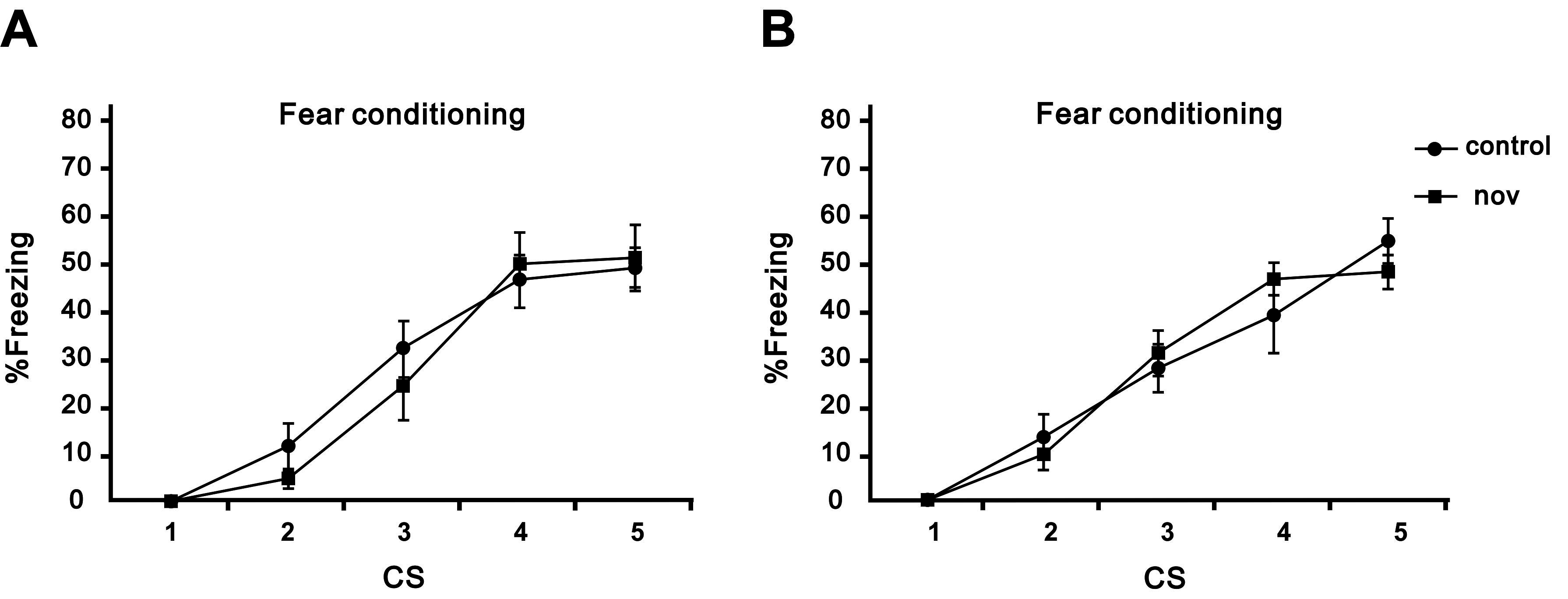


Supplementary Figure S1. All mice froze equivalently during auditory fear conditioning. (A) The control group (n = 7) and the nov group (n = 7) showed equivalent fear learning (*F* (1, 12) = 0.19, *p* = 0.67). Twenty-four hours later, they were submitted to extinction training, as mentioned in Fig. 1A. (B) The control group (n = 9) and the nov group (n = 10) showed equivalent fear learning (*F* (1, 17) = 0.005, *p* = 0.945). Thirty days later, they were submitted to extinction training, as mentioned in Fig. 1B.


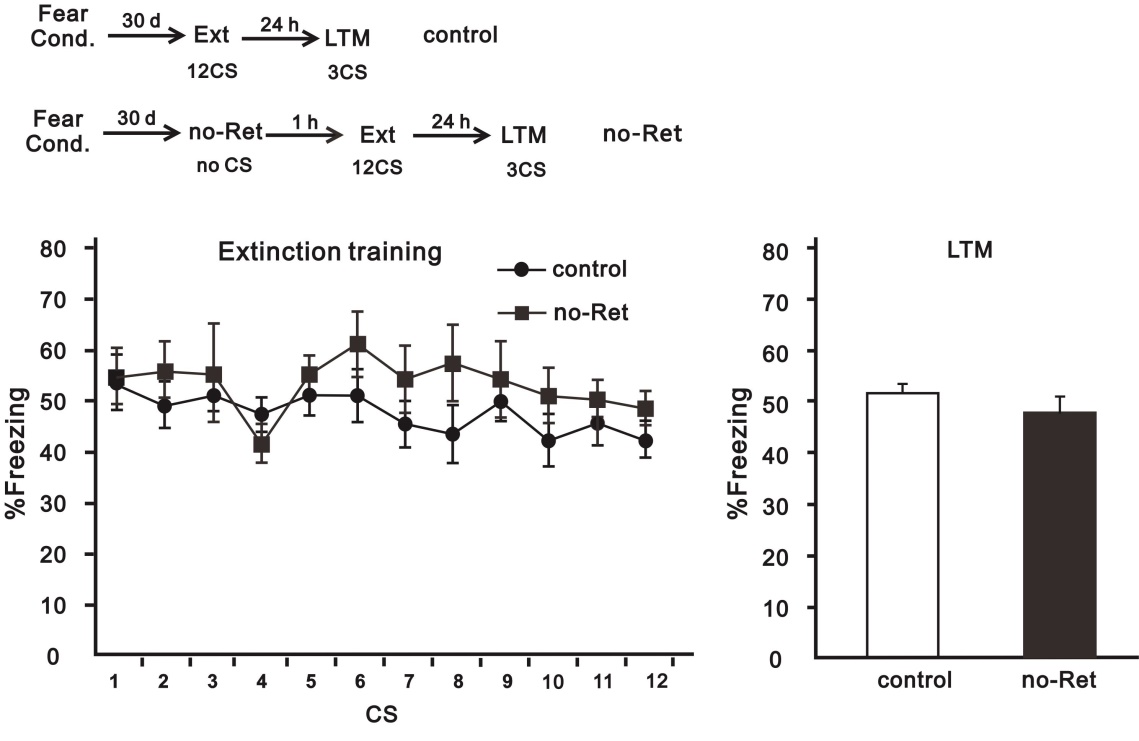


Supplementary Figure S2. Mice behaved similarly whether they were exposed to conditioning context (no-Ret) or simply left unexposed in the home cage (control) before extinction training. Mice were fear conditioned with five tone-shock pairings. After 30 days, they underwent an extinction training with 12 CSs. The no-Ret group were exposed to conditioning context for 4 min and 20 second, a time equal to the retrieval session, before extinction training. Twenty-four hours later, all mice were submitted to an LTM test session with 3 CSs. During both the extinction session (A) and LTM test (B), the control and no-Ret group exhibited similar fear response. Data are presented as mean ± SEM of the percentage of time spent freezing. n = 8 animals per group.


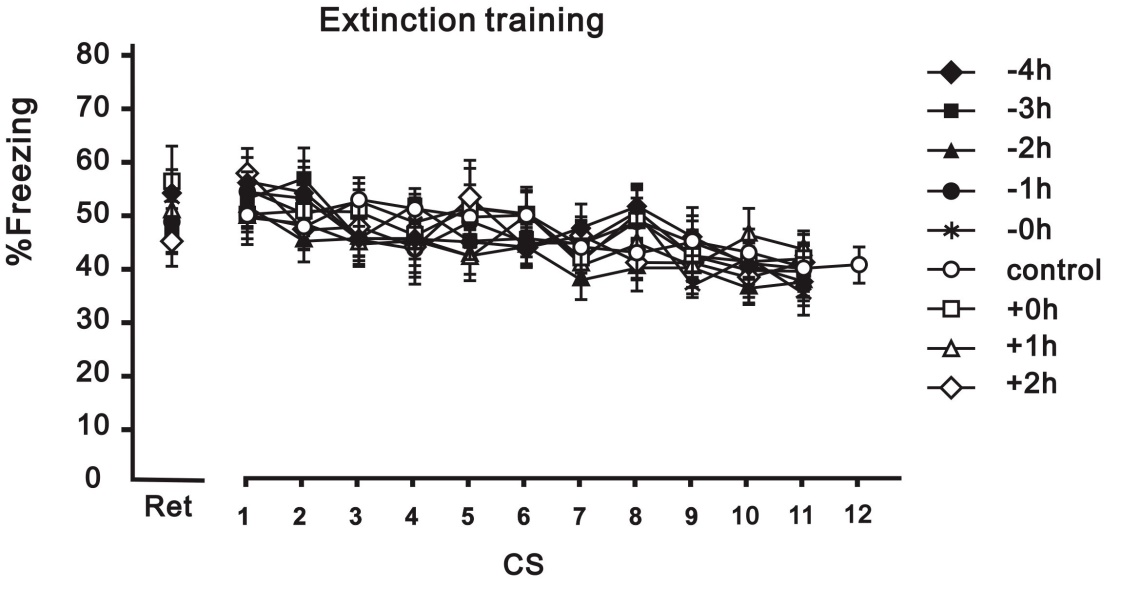


Supplementary Figure S3. Mice behaved similarly during extinction training, as mentioned in Fig. 1C.


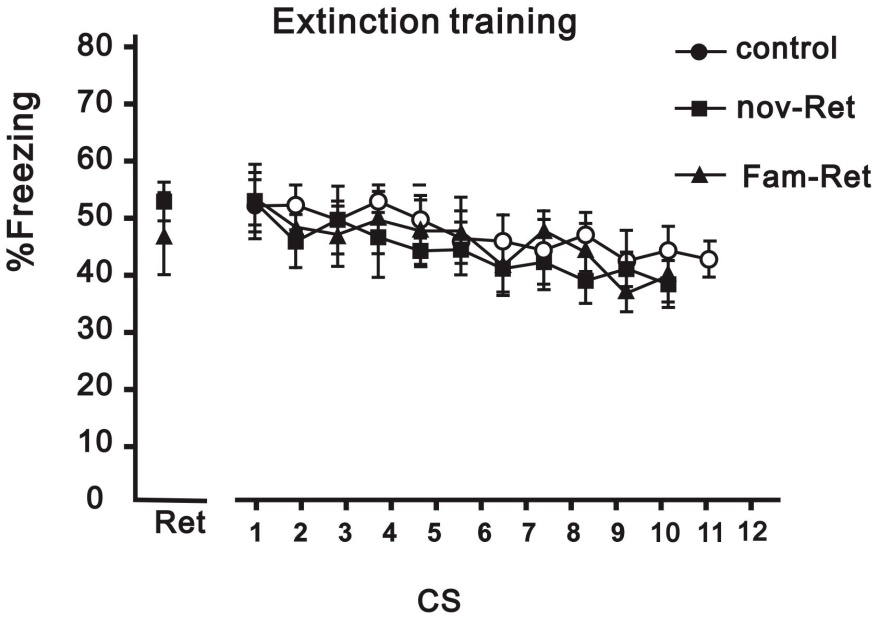


Supplementary Figure S4. Mice behaved similarly during extinction training, as mentioned in Fig. 1D.


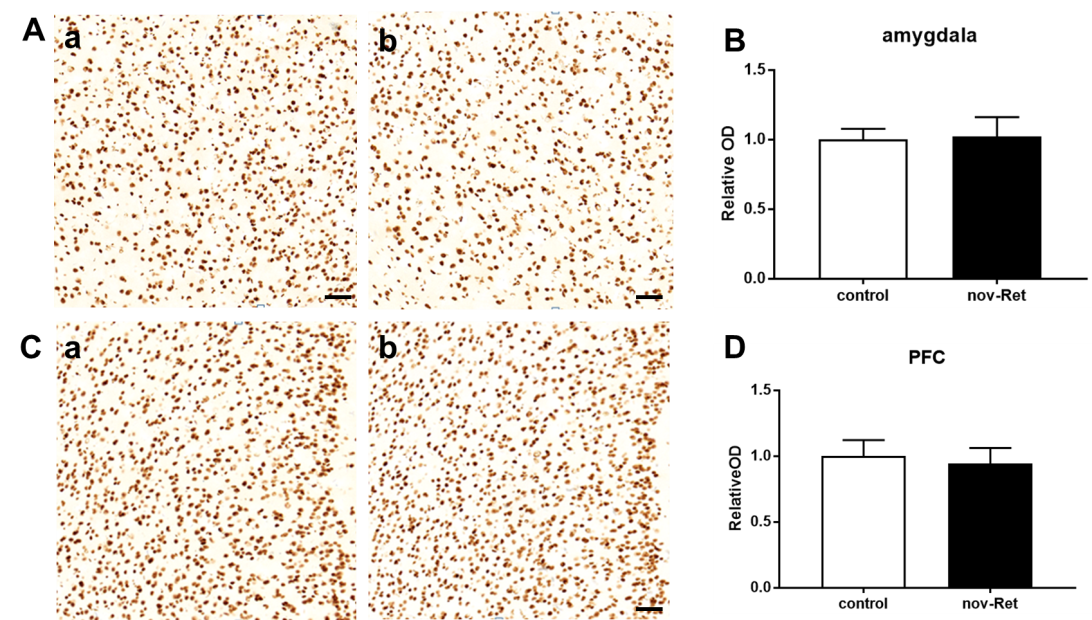


Supplementary Figure S5. Exposure to an OF before the retrieval-extinction session have no effects on H3K9/14 acetylation in PFC and amygdala.

(A) Representative images showing immunostaining for H3K9/14ac in the PFC region 1 h after completion of extinction in control (a) and nov-Ret mice (b). Scale bar: 100 μm. (B) Immunohistochemical analysis of OD for H3K9/14ac in the PFC for each group. n = 4 mice in each group. (C) Representative images showing immunostaining for H3K9/14ac in the amygdala region 1 h after completion of extinction in control (a) and nov-Ret mice (b). Scale bar: 100 μm. (D) Immunohistochemical analysis of OD for H3K9/14ac in the amygdala for each group. n = 4 mice in each group.


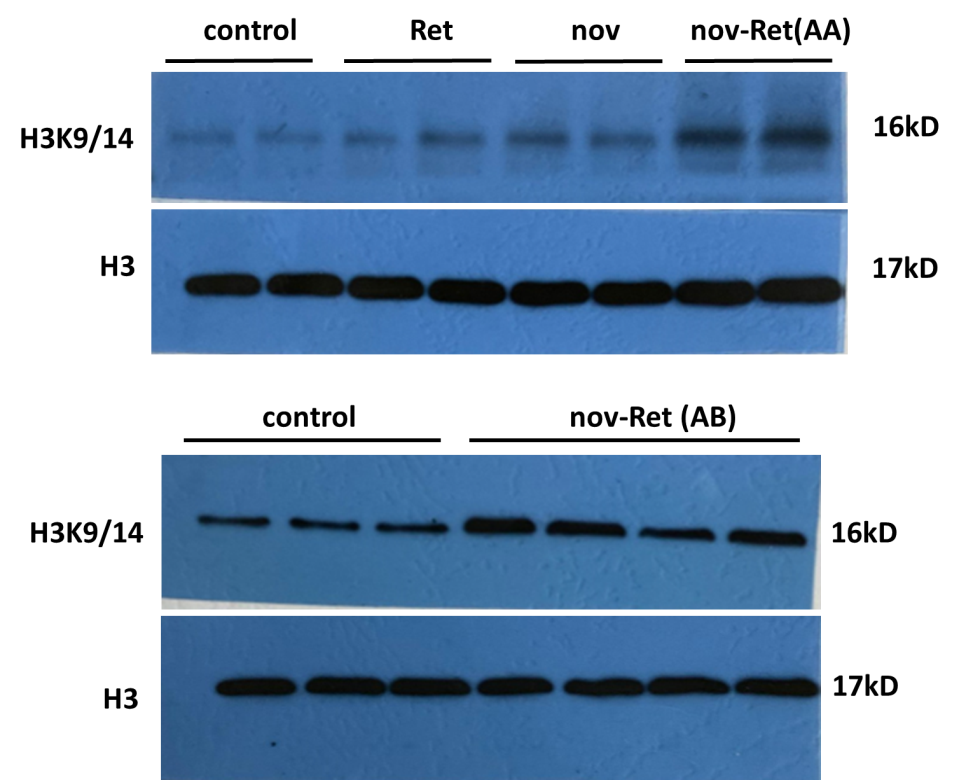


Supplementary Figure S6. Original pictures of western blot analysis of H3K9/14ac in the hippocampus 1 h after completion of extinction in control and nov-Ret mice. n = 4 mice in each group. Two mice in the nov-Ret group received fear conditioning and retrieval-extinction in the same context (AA), and another two in different contexts (AB).
